# Relating process evaluation measures to complex intervention outcomes: findings from the PACE-UP primary care pedometer-based walking trial

**DOI:** 10.1186/s13063-017-2428-z

**Published:** 2018-01-22

**Authors:** Cheryl Furness, Emma Howard, Elizabeth Limb, Derek G. Cook, Sally Kerry, Charlotte Wahlich, Christina Victor, Ulf Ekelund, Steve Iliffe, Michael Ussher, Peter Whincup, Julia Fox-Rushby, Judith Ibison, Stephen DeWilde, Tess Harris

**Affiliations:** 1 Population Health Research Institute, SGUL, London, UK; 2 Pragmatic Clinical Trial Unit, QMUL, London, UK; 3 Ageing Studies Research theme, Institute for Environment, Health and Society, Brunel University London, London, UK; 4 Department of Sports Medicine, Norwegian School of Sports Science, Oslo, Norway; 5 Research Department of Primary Care and Population Health, UCL, London, UK; 6 Department of Population Science, King’s College London, London, UK

**Keywords:** Process evaluation, Pedometer, Primary care, Walking intervention

## Abstract

**Background:**

The PACE-UP trial demonstrated positive effects of a pedometer-based walking intervention on objective physical activity (PA) outcomes at three and 12 months in 45–75-year-old primary care patients, in postal and nurse-supported trial arms compared with controls. We explored associations between process evaluation measures and change in PA outcomes.

**Methods:**

The MRC framework guided process evaluation. Three quantitative measures (nurse session attendance [dose delivered], PA diary completion [fidelity] and pedometer use [fidelity]) were selected as independent variables in multi-level models estimating intervention effectiveness on PA outcomes (changes in step-counts and time in moderate-to-vigorous PA [MVPA] levels in ≥ 10-min bouts).

**Results:**

Dose: attending all three nurse sessions compared with 0–2 sessions was associated with an increase in steps/day at three and 12 months of 1197 (95% confidence interval [CI] = 627–1766) and 605 (95% CI = 74–1137), respectively; and MVPA in bouts (min/week) at three and 12 months by 74 (95% CI = 45–103) and 30 (95% CI = 3–57), respectively. Fidelity: postal and nurse groups showed strong positive associations of diary return with steps/day at three months: postal 1458 (95% CI = 854–2061), nurse 873 (95% CI = 190–1555). MVPA in bouts (min/week): postal 64 (95% CI = 33–94), nurse 50 (95% CI = 15–85). At 12 months, only the postal group effects remained statistically significant: steps/day 1114 (95% CI = 538–1689), MVPA 47 (95% CI = 18–75). Regular pedometer use in the postal group only was associated with higher three-month and 12-month steps/day: 1029 (95% CI = 383–1675) and 606 (95% CI = 22–1190), respectively, and with MVPA in bouts at three months: 40 (95% CI = 6–73).

**Conclusion:**

Process evaluation measures demonstrated significant associations with PA outcomes at three and 12 months. We cannot infer causality, but the associations between the process measures and PA outcomes suggest that they were important in enabling the trial changes observed and should be considered core components of the PACE-UP nurse and postal interventions. We have shown the MRC framework to be a useful tool for process evaluation of intervention implementation.

**Trial registration:**

ISRCTN Registry, ISRCTN98538934. Registered on 2 March 2012.

## Background

The PACE-UP randomised controlled trial (RCT) is a complex intervention. RCTs establish intervention effectiveness, but do not tell us how or why an intervention works, and if it is not successful, why not. Process evaluation provides an assessment of the effective components of an intervention. Without evaluating the processes of the intervention, it is challenging to assess the validity of the contribution of an intervention to the research outcomes. This process evaluation investigates the relationship between the fidelity and quality of implementation, the context of the intervention and the main trial outcomes. The evaluation helped to illustrate replicability and generalisability of the intervention by relating process evaluation measures to objectively measured trial PA outcomes.

The MRC framework, developed in 2014 [1], built on the 2008 guidance [2] and on previous less comprehensive frameworks used to assess implementation fidelity alone (e.g. the modified conceptual framework [3] and RE-AIM [4]), offers the first useful tool to evaluate the entire process of a complex intervention. It can be used to assess fidelity and quality of implementation, clarify causal mechanisms and identify contextual factors associated with variation in outcomes.

Matthews et al. [5] used the 2014 MRC framework, in combination with guidance from Steckler and Linnan [6], the RE-AIM framework [4], and the World Health Organization [7], for process evaluation of a walking intervention; however, they did not clearly relate process and outcome measures. Van Bruinessen et al. [8] used the MRC framework to complete the PatientTIME web-based intervention process evaluation and showed significant improvements in perceived efficacy, but no significant association between process and outcome measures. Foley et al. [9] found no significant effect of the SWITCH study intervention on main trial outcomes, which could be related to the lack of fidelity in the intervention reported as a result of the process evaluation.

The PACE-UP trial is a large, pedometer-based, complex, walking intervention with two intervention arms (postal and nurse support) and multiple interacting intervention components (pedometer, handbook, physical activity [P]) diary, practice nurse PA consultations and behaviour change techniques [BCTs]). The aim of this process evaluation was to understand how the PACE-UP intervention was delivered and received and which intervention components were associated with the main positive trial PA outcomes.

## Methods

### PACE-UP trial design

PACE-UP was a three-armed RCT of a 12-week, pedometer-based, walking intervention with and without practice nurse support. In total, 1023 patients aged 45–75 years, from 922 households, with no contradictions to increasing PA were recruited from seven general practices in South London, UK, and randomised by household (one or two persons per household) to either control (*n* = 338), postal pedometer intervention (n = 339) or nurse-supported pedometer intervention (*n* = 346). The main trial outcomes were changes in average daily step-count and weekly time spent in moderate-to-vigorous physical activity (MVPA) (in ≥ 10-min bouts) between baseline and 12 months. The full study design, methods and outcomes are described in detail in the trial protocol [10] and outcome [11] papers. A brief summary of the PACE-UP intervention is provided, followed by the process evaluation methods used.

### The PACE-UP interventions

The intervention was designed to gradually increase step-count and MVPA over a 12-week period, with targets based on participants’ baseline physical activity levels. Participants’ physical activity was objectively measured using accelerometry over seven-day periods at baseline, three months and 12 months to assess main trial PA outcomes. The two intervention groups received pedometers, 12-week walking programmes, handbooks and PA diaries. The nurse group were additionally offered three PA consultations with a practice nurse at weeks 1, 5 and 9. The patient handbook and PA diary received by both intervention groups incorporated several BCTs adapted from the NHS Health Trainers Handbook [12]. Nurse sessions provided further BCTs, including individual goal-setting and motivational interviewing. Content delivered in the nurse-support arm was captured with nurse attendance logs completed by the nurses.

### Process evaluation

**Fig. 1:**
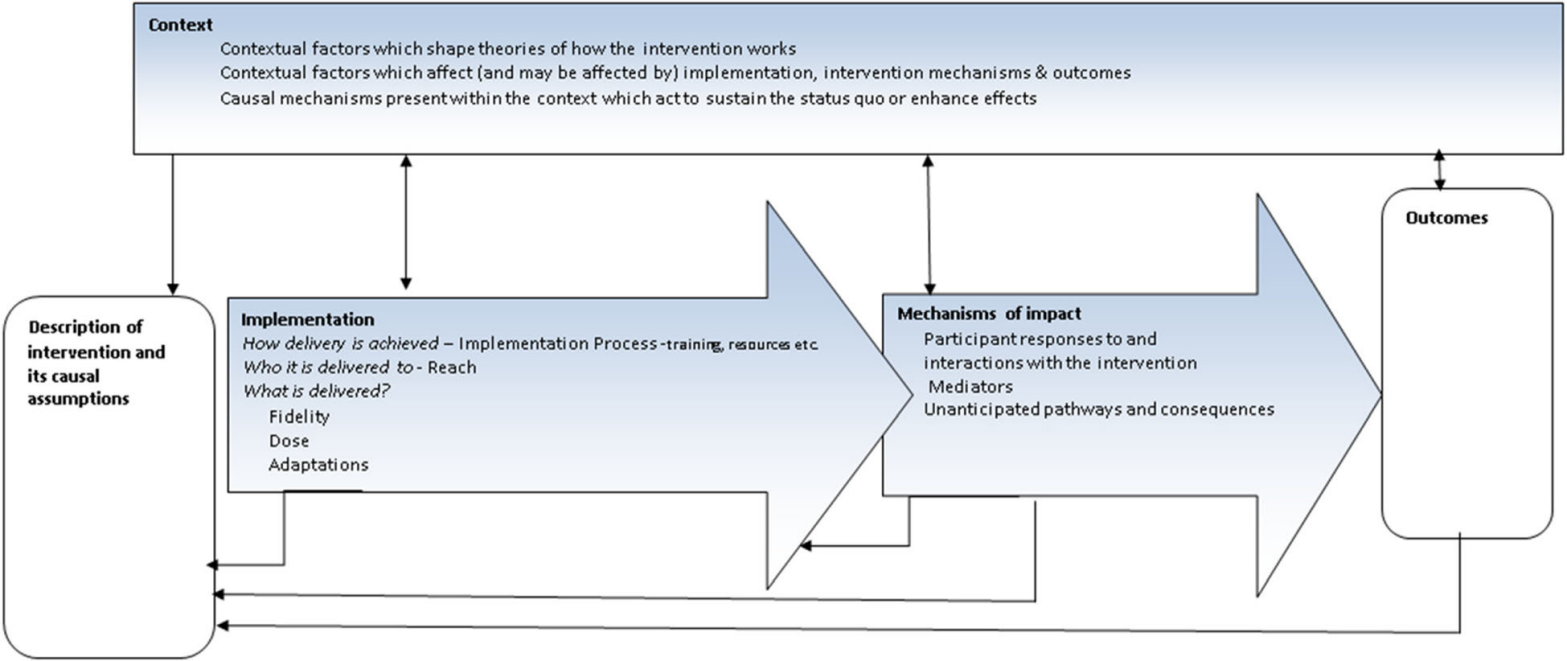
Key functions of process evaluation and relationships among them [1]. *Blue boxes* are the key components of a process evaluation. Investigation of these components is formed from intervention description and informs interpretation of outcomes

The process evaluation component of PACE-UP was designed in accordance with the MRC guidance framework 2014 [1]. Methods used were selected through the key functions model (Fig. 1). A mixed-methods (quantitative and qualitative) approach included assessment of all key functions: context; implementation (implementation process, reach, fidelity, dose, adaptations); and mechanisms of impact. Full details will be published in the Health Technology Assessment (HTA) report. Qualitative evaluations of participants [13] and practice nurses [14] are already published.

This paper focuses on implementation fidelity and dose in the nurse group, the dose delivered was fixed for the postal group. The evaluation included three key quantitative elements relating to implementation of the intervention: nurse session attendance (dose delivered); PA diary completion (implementation fidelity); and pedometer use during the 12-week intervention (implementation fidelity). Data sources and evaluation measures are identified and summarised in Table 1. Both intervention groups provided data on PA diary and pedometer use. However, dose delivered is only available for the nurse group with data on the number of nurse sessions attended.

**Table 1:**
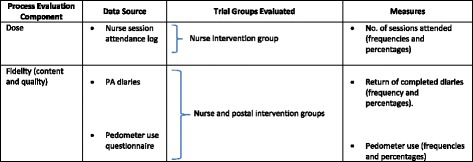
Components, data sources and measures for evaluating associations between process evaluation measures and trial PA outcome

### Data collection

#### Nurse session attendance: dose delivered

Dose delivered could fluctuate in the nurse intervention group depending on the number of sessions attended which could be in the range of 0–3 (the prescribed number). Data on the number of sessions participants attended were recorded by the nurses and allowed us to explore the relationship between dose delivered, 0, 1, 2, 3 sessions attended, and PA outcomes in this group at three and 12 months.

#### PA diaries: intervention fidelity

Twelve-week PA diaries were provided to both trial intervention groups and returned by participants to the trial team. The diaries provided data on daily step-counts and/or daily walks. A completed diary was defined pragmatically as one with three or more completed days for each of the 12 weeks of the intervention, to demonstrate that the diary had been used for the duration of the intervention. The relationships between completed diary return (Y/N) after the intervention (three months) and PA outcomes at three and 12 months were explored.

#### Pedometer use: intervention fidelity

Participants were asked about their pedometer use during the intervention period via a questionnaire completed at 12 months. Pedometer use was defined as how regularly the participant used the pedometer during the 12-week intervention period. The associations between pedometer use during the intervention (0–3 months) and PA outcomes at three and 12 months, respectively, were explored for both intervention groups using the response to the following question: How often did you wear the pedometer? The most positive responses were identified as reported pedometer use ‘every day’ or ‘most days’ and compared this with other less frequent responses.

### Analysis

The trial was powered for the analysis of the difference in PA outcome measures between the three trial groups and not for exploration of the effect of the process evaluation measures [10]. Nevertheless, it was important to explore the relationship between dose and intervention fidelity and change in PA outcomes in order to understand how different intervention components may have had their effects. To estimate change we regressed estimated average daily step-count at 12 months on estimated average daily baseline step-count, month of baseline accelerometry, age, gender, general practice and a group variable using the Stata command xtmixed. A household identifier was included as a random effect to account for clustering by household. The group variable was included to allow for treatment effects and to estimate differences in response between individuals within a treatment group, with different process measures. Thus, for number of nurse sessions attended, the group variable had the following four categories: Control; Postal; Nurse < 3 sessions; and Nurse 3 sessions attended. The lincomest post estimation command was then used to provide estimates and confidence limits for the difference between Nurse 3 sessions attended and Nurse < 3 sessions attended. Because we regressed 12 months on baseline step-count, the coefficients for the group variable is a direct measure of change from baseline. Similar analyses were conducted for different time points, MVPA and the other process measures. Full details of the accelerometry data processing and the statistical models used for the main trial outcomes are provided elsewhere [11].

## Results

### Main trial outcomes

The main trial outcomes have been published elsewhere [11], but are reported briefly here to provide context; in particular for the later comparison of the impact of process evaluation measures on trial outcomes. The PACE-UP trial recruited 1023 participants; 93% (*n* = 956) of participants provided accelerometry outcome data at 12 months. At three months, there were significant differences for change in step-counts from baseline between intervention groups and the control group: additional steps/day postal 692 (95% confidence interval [CI] = 363–1020), nurse support 1172 (95% CI = 844–1501). Findings for MVPA showed a similar pattern: additional MVPA in bouts (min/week) postal 43 (95% CI = 26–60), nurse-support 61 (95% CI = 44–78). At the main 12-month outcome, both intervention groups had increased their PA compared with controls; however, there were now no significant differences between the two intervention groups: additional steps/day postal 642 (95% CI = 329–955), nurse 677 (95% CI = 365–989) and additional MVPA in bouts (min/week) compared with control postal 33 (95% CI = 17–49), nurse-support 35 (95% CI = 19–51).

### Process evaluation measures

Table 2 shows the results for the process evaluation measures relating to implementation that we are using to model against PA outcomes. For dose, over three-quarters of participants in the nurse-support group attended all three nurse sessions, 95% (330/346) attended session 1 and 86% (296/346) attended session 2. Diary return was also high with overall 80% (549/685) returning their diaries and little difference between postal and nurse groups. Regular pedometer use was high during the intervention (0–3 months) in both intervention groups (85% overall); this was higher in the nurse-supported than the postal group.

**Table 2:** Nurse session attendance, diary return and pedometer use in the intervention groups^a^

	Postal (*n* = 339)		Nurse (*n* = 346)		Nurse + Postal (*n* = 685)	
Dose: attended all 3 nurse sessions	N/A		255	(74%)		
Diary return: yes	268	(79%)	281	(81%)	549	(80%)
Pedometer use during 12-week intervention: every day or most days	238	(81%)	269	(89%)	507	(74%)

^a^45 and 43 participants in the postal and nurse groups, respectively, did not answer the question on pedometer use during the 12-week intervention

### Relationship between process evaluation and PA trial outcomes measures

The modelling results relating nurse session attendance, PA diary return and pedometer use to PA outcomes (step-counts and time in MVPA in bouts) at three months 12 months are presented in Table 3.

**Table 3:** PACE-UP modelling results: relating nurse session attendance, step count diary return and pedometer use to PA outcomes

	Daily step count						Total weekly minutes of MVPA in ≥ 10-min bouts					
	3 months			12 months			3 months			12 months		
	Effect	(95% CI)	*P* value	Effect	(95% CI)	*P* value	Effect	(95% CI)	*P* value	Effect	(95% CI)	*P* value
*Nurse session attendance*												
Attended all 3 nurse sessions: YES vs NO	1197	(627–1766)	<0.001	605	(74–1137)	0.03	74	(45–103)	<0.001	30	(3–57)	0.03
*Step count diary return*												
Postal group: YES vs NO	1458	(854–2061)	<0.001	1114	(538–1689)	<0.001	64	(33–94)	<0.001	47	(18–75)	0.002
Nurse group: YES vs NO	873	(190–1555)	0.01	323	(−278–925)	0.29	50	(15–85)	0.005	3	(−27–33)	0.86
Nurse - postal difference	−585	(−1498–328)	0.21	−791	(−1624–42)	0.06	−14	(−60–33)	0.57	−44	(−85–-2)	0.04
*Pedometer use every day or most days during 12-week intervention*												
Postal group: YES vs NO	1029	(383–1675)	0.002	606	(22–1190)	0.04	40	(6–73)	0.02	26	(−2–55)	0.07
Nurse group: Yes vs NO	337	(−525–1198)	0.44	394	(−321–1109)	0.28	24	(−20–68)	0.28	10	(−25–45)	0.58
Nurse - postal difference	−692	(−1772–387)	0.21	−212	(−1136–712)	0.65	−16	(−71–39)	0.58	−16	(−62–29)	0.48

The nurse intervention group showed significant positive associations with dose of the intervention delivered (number of sessions attended) and step-count and time in MVPA in bouts at both three and 12 months. Participants attending all three sessions at three months increased their step-count significantly by 1197 steps/day (95% CI = 627–1766) more than those attending 0–2 sessions and at 12 months by 605 steps/day (95% CI = 74–1137). MVPA in bouts was significantly higher in those attending all three nurse sessions at both three months (74 min/week [95% CI = 45–103]) and 12 months (30 min/week [95% CI = 3–57]).

Both intervention groups at three months showed strong positive associations between diary return and PA outcomes. Diary return in the postal intervention group was associated with increased step count by 1458 steps/day (95% CI = 854–2061) and MVPA in bouts by 64 min/week (95% CI = 33–94) compared to those not returning a diary; and in the nurse intervention group an increase of 873 steps/day (95% CI = 190–1555) and 50 min/week of MVPA in bouts (95% CI = 15–85). At 12 months, only the postal group had sustained this significant association between diary return and increase steps of 1114 steps/day (95% CI = 538–1689) and 47 min/week (95% CI = 18–75) of MVPA in bouts with diary return. The directions of association were still positive in the nurse group at 12 months between diary return and both step-counts and time in MVPA, but the associations were no longer statistically significant and the differences between the nurse and postal groups were of borderline statistical significance.

Regular pedometer use in the postal intervention group (on either most days or everyday) during the intervention (0–3 months) showed strong significant increases in steps/day at three months by 1029 (95% CI = 383–1675) and at 12 months by 606 (95% CI = 22–1190) compared to those not reporting regular pedometer use; results were similar for MVPA (min/week) at three months with 40 (95% CI = 6–73) and in a positive direction, but not statistically significant at 12 months with 26 (95% CI = −2–55). Regular pedometer use in the nurse intervention group during the intervention was not, however, significantly associated with steps or time in MVPA at either three or 12 months, but nor were the effects significantly different from those in the postal group.

## Discussion

### Main findings

Our study results demonstrate the positive association between core components of the intervention and the main trial outcomes. For the nurse group, the number of sessions attended was significantly associated with both step-counts and time in MVPA at both three and 12 months. For both nurse and postal groups, return of a completed PA diary was positively associated with step-count and time in MVPA at three months and for the postal group at 12 months. Regular pedometer use was only associated with PA outcomes for the postal group (step-counts and MVPA at three months and step-counts at 12 months). While we cannot infer causality, the strong and consistent associations between nurse appointments, diary return and pedometer use identify these intervention components as important enabling factors in the main trial effects observed. The somewhat stronger impact seen in the postal group for diary return and pedometer use suggests that possibly the pedometer was a less important component for the nurse group. For the nurse group the number of nurse visits attended seemed to have a greater influence.

### Strengths and limitations

The study used data collected as part of the trial; this helped to reduce participant burden and optimise data completeness. We used data collected by both practitioners and participants, providing us with a more comprehensive assessment of the intervention. The data were collected longitudinally which allowed any change in intervention delivery over the course of the trial to be detected. The completeness of data sources strengthened the robustness of our findings.

The process evaluation was conducted by the trial team during the trial. This allowed efficient data collection, but as non-independent observers this could have led to bias in evaluation. Bias was minimised by the choice of data collection methods (e.g. nurse session attendance logs, completed by nurses), 12-month pedometer use questionnaires (completed by participants), and return of patient PA diaries, which was not influenced by the researchers. While data were collected from nurses and participants, the number of nurse sessions attended related more to participant responsiveness than to nurse engagement with the trial. This study was not powered to look at the effects of adherence to different aspects of the protocol on trial outcomes, which limits interpretation of these findings. Also, comparisons of those attending/not attending nurse appointments, those returning/not returning PA diaries and those using/not using pedometers were not randomised group comparisons, but within group comparisons, meaning that we can only describe associations between the process evaluation measures and trial outcomes, we cannot attribute causality. But our findings from the process evaluation on the value of attendance at nurse sessions and use of pedometer and diary are consistent with the qualitative finding from intervention participants [15] and practice nurses [16], which provided evidence that the pedometer, the recording of steps and PA in the diary and the nurse appointments were all important components of the trial and contributed to its success.

### Comparison with other studies

Process evaluation has become an increasingly important component of complex intervention Implementation and evaluation. Studies that have examined intervention implementation fidelity or process evaluation, have used a variety of frameworks and models as a structure to complete this which makes it difficult to draw direct comparisons with this study. Two studies reported analysis of the relationship between process evaluation outcomes and main trial outcomes [8, 9], but to our knowledge no trials have reported significant positive associations between process evaluation measures and main trial outcomes. A further novel feature of our study was the inclusion of perspectives from both nurse intervention delivery (number of sessions) and participant responsiveness (use of diary and pedometer); other studies report on a single perspective only and most commonly the person delivering the intervention [15–19]. For example, Van Bruinessen et al. reported participants’ perspectives and found that the intervention significantly improved perceived efficacy [8]; however, the association with outcome measures was not significant, therefore suggesting that the true effect of the intervention could not be identified. Berendsen et al. [20] evaluated both patient and practitioner perspectives, but demonstrated a much wider variation in dose delivered than we observed in PACE-UP, which makes it difficult to identify which of the intervention components were effective. They also reported problems with fidelity: healthcare professionals deviated from the protocol to reduce drop out from the trial, which had a positive effect on patient satisfaction [20]. Foley et al. [9] examined the relationship between process evaluation and trial outcomes in a weight loss trial; the intervention was not effective and investigators identified poor adherence to the intervention protocol as the most likely reason for this. The reduction in PA that we saw between three and 12 months, particularly in the nurse intervention group, could be seen as a case of ‘declining effect’ and directs attention to the maintenance part of the RE-AIM framework [4] and the whole question of intervention sustainability [21].

The PACE-UP intervention demonstrated that intervention dose (nurse session attendance) was associated with effectiveness of the intervention. Our finding of an association between return of a completed step-count diary and change in PA outcomes is consistent with the finding of a recent systematic review’s findings, which suggested that use of a step-count diary was common to many successful pedometer interventions [22]. We demonstrated that both intervention groups in the PACE-UP trial engaged well with the self-monitoring, using the pedometer and step-count diary. Although it is not possible to infer causality directly with the process evaluation data and the main trial outcomes, the high level of engagement with trial resources suggests that they were important influencing factors to make the PA changes observed. The associations between increased PA levels (steps and MVPA) and session attendance, step-count diary return and pedometer use emphasised that these components were active ingredients of the intervention. The MRC framework provided a useable logical and coherent structure for reporting [1]. The PACE-UP trial had a positive and significant effect on PA outcomes, but had this not been the case, the positive process evaluation with high levels of fidelity would have enabled us to have confidence that any negative trial effect would not have been due to poor trial implementation [3].

## Conclusion

The PACE-UP process evaluation demonstrated that the trial was delivered as per protocol with the MRC Framework a useful vehicle for reporting the evaluation. An association between several process evaluation measures and main trial PA outcomes has been demonstrated, suggesting that these components were important in effectiveness and should be considered core components of the PACE-UP nurse and postal interventions.

## Abbreviations

BCT: Behaviour change technique
CI: Confidence interval
MVPA: Moderate-to-vigorous physical activity
NHS: National Health Service
NIHR: National Institute for Health Research
PA: Physical activity
PACE-UP: Pedometer And Consultation Evaluation-UP
RA: Research assistant

## References

[1] Moore GF, Audrey S, Barker M, Bond L, Bonell C, Hardeman W (2015). Process evaluation of complex interventions: Medical Research Council guidance. BMJ..

[2] Craig P, Dieppe P, Macintyre S, Michie S, Nazareth I, Petticrew M (2008). Developing and evaluating complex interventions: the new Medical Research Council guidance. BMJ..

[3] Carroll C, Patterson M, Wood S, Booth A, Rick J, Balain S (2007). A conceptual framework for implementation fidelity. Implement Sci..

[4] Glasgow RE, Vogt TM, Boles SM (1999). Evaluating the public health impact of health promotion intervention: The RE-AIM framework. Am J Public Health..

[5] Matthews L, Mitchell F, Stalker K, McConnachie A, Murray H, Melling C (2016). Process evaluation of the Walk Well study: a cluster-randomised controlled trial of a community based walking programme for adults with intellectual disabilities. BMC Public Health..

[6] Steckler ALL (2002). Process evaluation for public health interventions and research.

[7] World Health Organization (2001). Process evaluation workbook.

[8] van Bruinessen IR, van Weel-Baumgarten EM, Gouw H, Zijlstra JM, van Dulmen S (2016). An integrated process and outcome evaluation of a web-based communication tool for patients with malignant lymphoma: randomized controlled trial. J Med Internet Res.

[9] Foley L, Mhurchu CN, Marsh S, Epstein LH, Olds T, Dewes O (2016). Screen Time Weight-loss Intervention Targeting Children at Home (SWITCH): process evaluation of a randomised controlled trial intervention. BMC Public Health..

[10] Harris T, Kerry SM, Victor CR, Shah SM, Iliffe S, Ussher M (2013). PACE-UP (Pedometer and consultation evaluation - UP) - a pedometer-based walking intervention with and without practice nurse support in primary care patients aged 45–75 years: study protocol for a randomised controlled trial. Trials..

[11] Harris T, Kerry SM, Limb ES, Victor CR, Iliffe S, Ussher M (2017). Effect of a primary care walking intervention with and without nurse support on physical activity levels in 45- to 75-year-olds: The Pedometer And Consultation Evaluation (PACE-UP) cluster randomised clinical trial. PLoS Med.

[12] British Psychological Society (2008). Improving health: changing behaviour: NHS Health Trainer Handbook.

[13] Normansell R, Smith J, Victor C, Cook DG, Kerry S, Iliffe S (2014). Numbers are not the whole story: a qualitative exploration of barriers and facilitators to increased physical activity in a primary care based walking intervention. BMC Public Health..

[14] Beighton C, Victor C, Normansell R, Cook D, Kerry S, Iliffe S (2015). “It’s not just about walking.....it’s the practice nurse that makes it work”: a qualitative exploration of the views of practice nurses delivering complex physical activity interventions in primary care. BMC Public Health.

[15] Ramsay CR, Thomas RE, Croal BL, Grimshaw JM, Eccles MP (2010). Using the theory of planned behaviour as a process evaluation tool in randomised trials of knowledge translation strategies: A case study from UK primary care. Implement Sci..

[16] Tomasone JR, Martin Ginis KA, Estabrooks PA, Domenicucci L (2015). Changing minds, changing lives from the top down: an investigation of the dissemination and adoption of a Canada-wide educational intervention to enhance health care professionals’ intentions to prescribe physical activity. Int J Behav Med.

[17] Tomasone JR, Arbour-Nicitopoulos KP, Pila E, Lamontagne ME, Cummings I, Latimer-Cheung AE (2017). Exploring end user adoption and maintenance of a telephone-based physical activity counseling service for individuals with physical disabilities using the Theoretical Domains Framework. Disabil Rehabil..

[18] Hasson H, Blomberg S, Dunér A (2012). Fidelity and moderating factors in complex interventions: a case study of a continuum of care program for frail elderly people in health and social care. Implement Sci..

[19] Grimshaw JM, Zwarenstein M, Tetroe JM, Graham ID, Lemyre L, Ecccles MP (2007). Looking inside the black box: a theory-based process evaluation alongside a randomised controlled trial of printed educational materials (the Ontario printed educational message, OPEM) to improve referral and prescribing practices in primary care in Ontario, Canada. Implement Sci.

[20] Berendsen BA, Kremers SP, Savelberg HH, Schaper NC, Hendriks MR (2015). The implementation and sustainability of a combined lifestyle intervention in primary care: mixed method process evaluation. BMC Fam Pract..

[21] Wiltsey Stirman S, Kimberly J, Cook N, Calloway A, Castro F, Charns M (2012). The sustainability of new programs and innovations: a review of the empirical literature and recommendations for future research. Implement Sci..

[22] Bravata DM, Smith-Spangler C, Sundaram V, Gienger AL, Lin N, Lewis R (2007). Using pedometers to increase physical activity and improve health: a systematic review. JAMA.

